# An overview of therapeutic applications of ultrasound based on synergetic effects with gold nanoparticles and laser excitation

**DOI:** 10.22038/ijbms.2019.29584.7142

**Published:** 2019-08

**Authors:** Ahmad Shanei, Ameneh Sazgarnia

**Affiliations:** 1 Medical Physics Department, School of Medicine, Isfahan University of Medical Sciences, Isfahan, Iran; 2 Medical Physics research Center, Mashhad University of Medical Sciences, Mashhad, Iran; 3 Department of Medical Physics, Faculty of Medicine, Mashhad University of Medical Sciences, Mashhad, Iran

**Keywords:** Cavitation, Gold nanoparticle, High intensity focused- ultrasound, Laser excitation, Sonodynamic therapy, Therapeutic ultrasound

## Abstract

Acoustic cavitation which occurs at high intensities of ultrasound waves can be fatal for tumor cells. The existence of dissolved gases and also the presence of nanoparticles (NPs) in a liquid, irradiated by ultrasound, decrease the acoustic cavitation onset threshold and the resulting bubbles collapse. On the other hand, due to unique capabilities and optical properties of gold nanoparticles (GNPs), they have been emphasized as effective NPs in the field of tumor therapy. Absorption of the laser light by GNPs causes the water molecules around the NPs to evaporate and produces vapor cavities. In this paper, we have reviewed published studies in the fields of ultrasound therapy, sonodynamic therapy (SDT) and synergism of low-level ultrasound and also laser radiation in the presence of GNPs.

## Introduction

In recent years, different methods of tumor treatment by ultrasound waves have been successfully developed. Applications of therapeutic ultrasound are based on its interactions with tissues, which create biological effects (1). 

High intensity focused ultrasound (HIFU) is a non-invasive method which has the potential of treating target tissues within a small focally selected volume (2). The focused ultrasonic beam produces heat to destroy target cells without creating harmful effects on the overlying or surrounding normal tissues (3). Thus, ultrasound waves penetrate through soft tissues and generate localized high temperatures in a few seconds (2, 3). Therefore, the primary mechanism in tumor treatment by HIFU is its thermal effect (3). 

Alternatively, the biological effects and therapeutic applications of low-intensity ultrasound (LIU) on tumor cells are under investigation. 

There is evidence showing that the responses of tumor cells to LIU are more severe than normal cells, in other words, tumor cells are more sensitive to LIU than the normal ones (4, 5).

In recent years, the therapeutic applications of LIU have generated an expanding field. The trace of such studies can result in improving chemotherapy and drug delivery (6). Ultrasound waves can be used to activate some anticancer agents in SDT (7). The mechanisms of SDT in inducing antitumor effects are mediated by cavitation (8). The SDT agents typically have low toxicity, and their activation by ultrasound waves generates free radicals (9).

On the other hand, chemotherapy can play an essential role in cancer treatment; however, administration of chemotherapy anticancer drugs should be limited due to potential side effects on healthy tissues (6).

Researchers have look into improving treatment methods of malignancies, in the meantime reducing their side effects (6). Enhanced lethality of anticancer agents with ultrasound exposure has made it possible to apply a lower drug dosage and at the same time increase the patient’s tolerance to chemotherapy (6). Moreover, ultrasound waves have an important role in the transfer of therapeutic agents into the target tissue (10). Created microbubbles are capable of producing forces that permeabilize cell membranes and carry therapeutic agents into the cells (10). 

Biological effects of ultrasound are mainly caused by heat, mechanical effects, and cavitation. Among the mentioned effects, inertial cavitation is the most important biological effect of ultrasound (11). There are two types of cavitation: stable cavitation and inertial cavitation. In stable cavitation, the bubble diameter changes about an equilibrium value, determined by the ultrasound wave’s frequency (12). In inertial cavitation, bubbles expand up to 2–3 times their resonant size and finally collapse in a compression half-cycle (13, 14). 

Considering ultrasound waves potential for inducing biological effects via drug delivery, inertial cavitation is considered as the most important property, by its non-thermal mechanisms (15). 

Although theoretical principles have suggested the occurrence of biological damage is due to stable cavitation bubbles (16), it is generally accepted that inertial cavitation is the most important mechanism for producing both irreversible damage (11) and increase in cell membrane permeability, which creates structural alteration of intact cells (17).

The main concern of researchers on increasing the therapeutic efficiency of ultrasound is to provide target tissue selectivity and to reduce the ultrasound intensity, needed for the occurrence of inertial cavitation.

On the basis of a few reports, the existence of a particle in a liquid provides a nucleation site for the cavitation bubble because of its surface roughness and bubble collapse. There are two procedures to use GNPs, as stated below:

1- Using nanoparticle as a nucleation site to decrease the intensity threshold needed for cavitation, and increase the number of bubbles and their resulting collapse (18). 

2- Depositing the laser light on the GNPs, leads to vaporization of the surrounding medium around the GNPs and formation of vapor cavities (19).

 In the present paper, we have provided information encompassing the mechanisms of action of ultrasound therapy, SDT, and synergism of ultrasound and laser radiation in the presence of GNPs.

***The role of NPs in increasing efficiency of ultrasonic treatments***

In recent years, ultrasound interactions with biological tissues have been extensively studied, and certain induced structural and/or functional alterations have been confirmed (12, 13). According to these investigations, most of the effects result from chemical, thermal, mechanical and optical procedures. However, the most important mechanical effect is acoustic cavitation (20). 

Among interactions of ultrasound with biological tissues, the thermal effect and cavitation are relatively well-understood (20). Biological effects of ultrasound waves depend on the ultrasonic frequency (13). The thermal effect is caused by the absorption of ultrasound into biological tissues. Ultrasound waves cause vibration or rotation of molecules or part of macromolecules in the tissue, and this movement results in frictional heat. The thermal effect caused by acoustic cavitation is larger than that caused by ultrasound absorption alone (21).

Hyperthermia is defined as the physiological increase in tissue temperature. Under hyperthermic conditions, depending on the temperature and the duration of exposure, the tissue may become more sensitive to chemotherapy or radiotherapy (20, 21).

For hyperthermia induction, HIFU is utilized (21). HIFU, produced by focalized transducers, can be applied either as the demolition of the tumor cells or as palliative therapy (22). HIFU has been utilized to treat solid tumors, and investigators have confirmed its efficacy (2, 13). The biological effects of HIFU were investigated in 1927 (23).

Some investigation showed that focusing ultrasound waves produces localized biological effects (24, 25). 

The first clinical application of HIFU for treating prostate cancer was reported in 1994 (26), and then utilization in various organs followed. HIFU potentials in medical applications are thermal ablations, thrombolysis, and targeted drug and gene-delivery systems (26, 27). During HIFU treatment, other mechanisms such as cavitation may take part beside hyperthermia in the tissue-damaging process (28).

Cavitation is one of the most important varieties of ultrasound bioeffects (29). It has been experimentally shown that the collapsing cavity produces high temperatures (up to 5000 °K) in tissues and increases the pressure (up to 1800 atm) for an extremely short period in micron dimensions (30, 31). Furthermore, the collapse of bubbles causes strong physical effects in the surrounding environment of bubbles which creates chemical and biological effects (32, 33). 

There are different methods, such as sonoluminescence (SL) and acoustic imaging, which are applicable in quantifying the cavitation. 

In 1991, researchers studied the acoustic cavitation onset threshold and bubble collapse in agar gel. They indicated that many features of the production of these bubbles could be described qualitatively (16). 

When bubbles collapse in a liquid such as water, imploding bubbles produce a significant enhancement in the localized temperature that in turn, leads to the breakdown of water molecules and the formation of hydroxyl radicals (34). 

Equations for production of hydroxyl and hydrogen free radicals by bubble collapse in water and decomposition of water has been described by Fang *et al*. (35). These chemical products were measured to detect cavitation activity.

Terephthalic acid (TA) is a suitable dosimetric solution for measuring the number of free hydroxyl radicals created by bubble collapse (36). When TA solution is irradiated by ultrasound waves, it generates hydroxyl radicals through water sonolysis (37). Then, the TA solution reacts with hydroxyl radicals, and 2-hydroxyterephthalic acid is produced, which can be detected using fluorescence spectroscopy (38). 

The main factor affecting acoustic cavitation is the existence of small stable gas bubbles that act as cavitation nuclei (15, 17). The number of available nuclei increases cavitation activity, which is ubiquitous in nondegassed liquids but is considered rare in tissues (15). Available physical space for growing bubbles is an important factor of formation, too. Therefore, cavitation induction within undamaged cells and in the extracellular matrix seems to be a complicated process (39). On the other hand, the vasculature has both the required space and cavitation nuclei, in the event that the ultrasound intensity the negative pressure peak of the ultrasound exceed the acoustic cavitation onset threshold (40). 

Recently, the application of cavitation-assisted therapy has been widely investigated. 

A study explored the cavitation in the presence of microbubbles, which was applied to induce tissue ablation and ultrasonic surgery (41).

An *in vivo* observation further clarified the cavitation activities and the cavitation-induced tissue damage; it implied that microbubble-assisted cavitation could cause tissue damage in a tiny region (42). Also, it is shown that microbubbles can improve ultrasound-mediated drug delivery and cell rupture (43, 44). 

There are a few reports that suggest the existence of dissolved gases and nanoparticles (NPs) in a liquid provides the nucleation sites for the cavitation during ultrasound exposure. They anticipate this finding is an outcome of the particles’ surface roughness, significant decrease of the acoustic cavitation onset threshold, and an increase in the number of bubbles (36).

In 1991, a study reported that a decrease in acoustic cavitation onset threshold could occur with increasing suspended particle concentration in liquid (45).

Chen *et al*. believe that cavitation increases in the presence of the microparticles due to increasing the number of cavities in the liquid (46).

In 2005, it was indicated that alumina particles with an appropriate amount and size increase the output of sonochemical reaction in aqueous KI solutions (18). They found a relation between the acoustic cavitation and oxidation reaction index of a KI solution during ultrasound irradiation in the presence of alumina particles (18). 

The cavitation potential was investigated via SL detection and TA chemical dosimetry methods at therapeutic intensities of ultrasound (34). In that research, SL was monitored on agar gel phantoms. When the agar gel phantom is exposed to ultrasound waves, cavitation may occur in the gel, and this leads to free radical production. This results in the production of SL emission (34).

In 2013, an *in vivo* investigation approved the acoustic cavitation in the presence of GNPs as an approach for improving tumors therapeutic effects (47). Their results showed that ultrasound irradiation alone has no significant antitumor effects, but its effects are enhanced by ultrasound waves in the presence of GNPs (47). In their study, ultrasound exposure was performed by a planar transducer without any significant increase in media temperature, indicating that the therapeutic effects were not related to hyperthermia. Therefore, it was anticipated that cavitation might have acted during ultrasound irradiation (47). 

A significant decrease in the tumor volume was reported, eight days after treatment of the mice irradiated using ultrasound in the presence of GNPs compared to those in the control group. The same results were reported for the above mentioned treated group and the groups that received GNPs or ultrasound alone, which confirms the effect of nucleating cavitation in the presence of GNPs (47).

Regarding *in vivo* investigations, certain limitations related to GNPs administration have been applied in order to arrange their distribution in tumors selectively. 

In 2008, researchers showed that GNPs’ biodistribution is size dependent. They showed that NPs which are smaller than 10 nm are distributed widely in different organs including the heart, lungs, liver, and also in the blood flow. (48).

Other researchers revealed that NPs could be accumulated in some structures, such as the liver and spleen, for a long time post-intravenous injection regardless of their size, shape, and dose.

In 2010, rapid biodistribution of GNPs and also changes of gene expression in the liver and spleen in rats after intravenous injection was shown (49). It should be noted that a lower dose of GNPs can be injected to overcome some side effects intratumorally (47).

Researchers evaluated acoustic cavitation by the TA chemical dosimetry method in the presence of GNPs and revealed that the fluorescence signal for TA solution in the presence of GNPs is higher than the TA solution in the absence of GNPs in different intensities of ultrasound in the continuous mode (36). They suggested that these results could be related to the following chain: (1) GNPs acted as cavitation nuclei and increased the cavitation rate (2) and GNPs increased the collapse of cavities (19). 

GNPs’ oscillation during ultrasound irradiation should not be forgotten. In other words, the particles may act as the new wave sources for which these waves may interact with the bubbles, induced by the main ultrasonic waves, and cause them to collapse. Another possibility can be the bubbles’ impact on the GNPs’ surface resulting in the destruction of the bubbles. 

The effect of GNPs in different sizes on the cavitation activity have been investigated by detecting and quantifying free hydroxyl radicals in TA solutions containing GNPs in different sizes by using 1 MHz low-level ultrasound (50). In that research, TA solution was also utilized as a chemical dosimeter to quantify the free hydroxyl radicals generated by the collapse of the inertial cavities resulting from low-intensity ultrasound. This dosimetry is based on the fluorometric method, which is very sensitive to hydroxyl radical measurement.

In that study, it has been shown that the number of cavitation bubbles is increased with a rise in the size of GNPs, which results in an enhancement of the number of hydroxyl radicals (50). 

One of the therapeutic applications of ultrasound waves is SDT (51). SDT refers to the ability of ultrasound waves to produce cytotoxic effects on different cell lines (52). The cytotoxicity of SDT can be increased in the presence of drugs that are sonosensitizers. Ultrasound waves can be focused on the tumor and activate the sonosensitizing drug (53, 54).

In 1994, investigators suggested that SDT cytotoxicity is mediated by inertial cavitation (55). During inertial cavitation, a gas bubble is created by ultrasound. After a rapid collapse, a shock wave is produced by releasing an intense heat (56). These events cause pyrolysis of the surrounding water molecules and their decomposition into ^°^H and ^°^OH radicals, which either recombine or reduce solute molecules, such as sonosensitizing agents or the biomolecules (56).

Several researchers have reported that ultrasonic cavitation produces free radicals, such as hydroxyl, singlet oxygen, and hydroperoxyl. These molecules have an important role in inducing the synergistic effects of ultrasound and sonosensitizers (57).

In the past two decades, increased attention has been attracted to the role of reactive oxygen species (ROS) in experimental medicine (58). ROS is generated by all aerobic organisms. Intracellular production of ROS threatens the integrity of macromolecules involved in DNA synthesis and also induces damage to mitochondrial DNA (59). The presence of unpaired electrons in free radicals usually increases reactivity. 

*In vitro* investigations have shown that SDT leads to cell lysis in erythrocytes, sarcoma 180, L1210, and HL-60 (53). *In vivo* investigations have revealed that this type of treatment can be useful in treating colon-26 carcinoma (7, 8). 

***Enhancement of SDT efficacy in the presence of GNPs***

Researchers are looking for an effective method for targeting tumors with minimal side effects to normal tissues (60). 

One of the advances in SDT is the introduction of conjugated antibodies in the process of targeted delivery of sonosensitizers. In this technique, a monoclonal antibody is connected with the sonosensitizer, so that they can be specifically linked to the target cell membrane. During the sonication process, the target cells are destroyed efficiently, which makes the treatment more effective (61). Therefore, the synergism of the following three factors in SDT is essential for converting this treatment into an effective approach in tumor therapy: selection of a high yield sonosensitizer, its targeted delivery to the tumor, and its activation by LIU. Moreover, most sonosensitizers are fluorescent, and this property can be utilized in order to better trace and localize them. Therefore, sonosensitizer localization in target tissues can be evaluated before the application of ultrasound to the tumor.

Animal and cellular studies have shown that ultrasound can induce anti-tumor effects via activating certain porphyrins (53, 62).

Also, chemical activation of some hematoporphyrins by acoustic cavitation was studied by researchers*.* According to their suggestions, this activation could be due to HpD pyrolysis or reactions with OH^°^ radicals (63). 

Other investigators stated that the photosensitizers are not activated by sonoluminescence (53).

An investigation showed the relation of the ultrasound-induced decomposition of porphyrin with the rise in the NO_3_^-^ and H_2_O_2_ concentrations in the medium (64). 

In 1993, after comparing the effect of hematoporphyrin with that of ATX-70 in sarcoma of 180 different cells, it was suggested that the structure of the sonosensitizer influences the efficiency of SDT (62).

Researchers have reported, their findings on the damage of deoxyribonucleic acid (DNA) after SDT in the presence of hematoporphyrin-gallium (HP-Ga) (65). These damages were enhanced with increasing ultrasonic irradiation time and HP-Ga complex concentration (65). 

Protoporphyrin IX (PpIX) is an efficient hydrophobic sensitizer with both properties of photo- and sonosensitizing (66). Activated PpIX produces cytotoxic reactive oxygen species via interacting with molecular oxygen, and subsequently causes irreversible destruction of the the target tissue (67). 

In 2007, SDT in the presence of PpIX disodium salt on S180 solid tumors was studied (68). In order to determine the optimum time interval between administration of PpIX and ultrasound exposure, PpIX concentration in plasma, skin, muscle, and tumor was assessed (68). *In vivo *antitumor effects of SDT were caused decreasing tumor size, increasing survival time of the tumor-bearing mice, and also, the morphological changes of the S180 cells (68). Their findings suggested a 24 hr interval between administrating PpIX and irradiation, as the optimum time for ultrasound exposure (68). 

In the innovative investigations, nanostructures that can be made hydrophobic, have been utilized to improve anticancer therapeutic approaches (69). Their enormous surface area can be increased by using functional groups possessing a variety of biochemical or chemical properties arrays. Due to their subcellular size, NPs can go through the tissues and then be taken up efficiently by cells (18). In the meantime, some specific characteristics of GNPs including special optical properties, low toxicity and good uptake by mammalian cells have made them highly attractive for medical applications. Nowadays, GNPs are used for targeted drug delivery and as tumor sensitizers (69).

On the other hand, one of the existing challenges in SDT is the use of high-intensity ultrasound. This is mainly because of the reliant activation of the sonosensitizers with the cavitation. Furthermore, high-intensity ultrasound can also cause bio-effects on healthy tissues (70). It has been shown that the presence of NPs in a liquid provides a nucleation site for generating bubbles and decreases the needed intensity threshold of ultrasound for cavitation (71). Also, it is anticipated that the surface roughness of the particles helps bubbles collapse (69). 

Comparing the non-radiant relaxation time between Au-PpIX and PpIX, it can be stated that the non-radiant relaxation time of sonosensitizer in the presence of GNPs is longer than the one without GNPs (72). The longer non-radiative time provides singlet oxygen more efficiently. 

Researchers suggested the use of a novel designed sensitizer to reinforce SDT efficacy. Moreover, they have studied the role of PpIX in inducing *in vivo* sonodynamic antitumor effects (69).

In that study, ultrasound irradiation alone did not show significant antitumor effects, which was increased by ultrasound in the presence of PpIX. The inhibitory effect was significant when ultrasound together with Au-PpIX conjugate was used. It was found that conjugated PpIX to GNPs promoted the antitumor effects of SDT (69). 

In 2011, investigators studied SDT using PpIX conjugated to GNPs (Au-PpIX) (73). They reported that the best response to treatment appeared in the ultrasound with Au-PpIX group.

 It should be noted that the size of the GNPs plays an important role in inducing biological effects (73).

The induction of cytotoxicity through sonosensitization in tissues is created by singlet oxygen molecules (74). The second results could have been related to several stages of action: (1) facilitating the entrance of PpIX into the tumor cells GNPs (75), (2) the role of PpIX as the sonosensitizer and the GNPs as cavitation nuclei (71) and also (3) the increase in cavities collapse.

A researcher has reported that the chemical activation of sonosensitizer leads to sonosensitization, happening in the close vicinity of hot collapsing cavitation bubbles. According to their findings, this process has formed sensitizer-derived free radicals in high-energy states (63). This energy level can be transferred to the PpIX molecules in order to form excited-state PpIX. The energy transfer from activated PpIX to an oxygen molecule produces singlet oxygen (76).

Other findings suggest that the Au–PpIX conjugate has greater potential than PpIX alone as a sonosensitizer in the treatment of tumors by SDT (69). *In vivo* studies revealed a significant inhibitory tumor growth for SDT with the GNP and PpIX conjugate. This has been known due to the increased uptake of PpIX by the cells, which could be the result of the presence of GNPs as carrier and also cavitation nuclei. Although tumor growth could be inhibited by ultrasound alone and SDT using PpIX (69), such findings have also been confirmed by other similar studies performed on phantoms. 

In 2011, researchers investigated sonochemiluminescence (SCL) in a polyacrylamide gel phantom containing luminol in the presence of PpIX conjugated nanoparticles (77). They showed that the SCL signal in phantoms containing Au–PpIX is higher than in the other phantoms. This could be related to the existence of PpIX as a sonosensitizer and GNPs as the cavitation nuclei (77). 

The capability of GNPs in converting light to heat is one of their other unique features. This photothermal property is used for vaporizing cell water that in turn will reduce the ultrasound intensity threshold for the cavitation. Comparison of the properties of different studies on GNPs is shown in Table 1.

**Table 1:** Comparison of the properties of different studies on GNPs

Type of study	Type of NP	Concentration and size of NP	Study method efficacy evaluation	Reference
Polyacrylamide gel phantom	GNPs	0.22 5-9 nm	Sonochemiluminescence and detecting free hydroxyl radicals	29
Agar gel phantom	Au-PpIX	0.39 mg/ml 7 nm	Sonoluminescence	34
Terephthalic acid dosimetry	GNPs	0.22 mg/ml 5-9 nm	Detecting and quantifying free hydroxyl radicals	36
*In Vivo* study on a colon tumor model	GNPs	0.22 mg/ml 6-8 nm	Measurement of tumor diameters, doubling time, etc.	47
*In Vivo* study	GNPs	10, 50, 100 and 250 nm	Inductivity coupled plasma mass spectrometry	48
Terephthalic acid dosimetry	GNPs	20, 40, 60 and 80 mg 15, 20, 28 and 35 nm	Detecting and quantifying free hydroxyl radicals	50
*In Vivo* study on a colon tumor model	Au-PpIX	0.22 mg/ml 7 nm	Measurement of tumor diameters, doubling time, etc.	69
Polyacrylamide gel phantom	Au-PpIX	0.39 mg/ml 7 nm	Sonochemiluminescence	77

***Synergism of ultrasound and laser radiations in the presence of GNPs***

In recent years, the interaction between laser radiation and metal NPs has been studied by many scientists, due to their unique photoacoustic and chemical properties. 

There are several reasons for this distinctive phenomenon. Photothermal effects are induced by using laser light at a certain wavelength that is absorbed by the NPs and with a pulse duration that is short enough to minimize heat leakage away from the absorbing particles. The laser light absorption is used in different photophysical and photochemical processes (78). In particular, GNPs have been studied for a variety of applications. Today, the optical and thermophysical properties of GNPs are of fundamental interest (78). 

The existence of NPs and gas dissolved in a liquid irradiated with ultrasound waves provides a nucleation site for cavitation and may decrease threshold intensity for the cavitation, and is also responsible for increasing the number of bubbles (18). 

As the pre-existing nucleation sites of cavitation are not omnipresent in most tissues, ultrasonic contrast agents have been investigated as providers of the nuclei (79), but they have a short life span. Laser irradiation in the presence of minute optical absorbers can be considered as an alternative to ultrasonic contrast agents (19). In this regard, some studies conducted on tissue-equivalent materials, have shown that absorption of short laser pulses by the NPs provides vaporization of water in the surrounding medium and the formation of transient vapor cavities (19). 

In 2005, it was reported that vapor bubbles generated by laser-illuminated GNPs in an acrylamide phantom provide nucleation sites for inducing cavitation by HIFU and have a remarkable role in decreasing the cavitation threshold intensity (19).

In 2011, monitoring of inertial cavitation induced by ultrasound and IPL in the presence of GNPs in a polyacrylamide gel phantom containing luminal was investigated (29). When the gel was irradiated by ultrasound, inertial cavitation occurred in the gel. Following the collapse of the bubbles, free radicals were produced, and then the SCL was emitted from the chemical reaction of luminol molecules with OH radicals produced by inertial cavitation (29). Their results showed that the SCL signal in gel phantoms containing GNPs was higher than in the gel phantoms without GNPs (29). As observed in the mentioned research, the highest integrated SCL signals were recorded in gel phantoms containing GNPs in the presence of IPL (29). The results have also been confirmed by TA dosimetric data (36). 

In some studies, the therapeutic effects of cavitation were examined in the presence of GNPs and intense pulsed light (IPL) to enhance the therapeutic effects on tumor models (69). According to their results, ultrasound individually showed insignificant antitumor effects, which was increased in the presence of GNPs. The inhibitory effect was significant when IPL and ultrasound together with GNPs were applied. It was anticipated that IPL irradiation on GNPs promoted the antitumor effects via providing nucleation sites for ultrasonic cavitation (69). 

Both the doubling time and 5-folding time can be good indicators for evaluating tumor growth (69, 80). The research results has shown more prominent inhibitory effects, shorter doubling and 5-folding times, and also delayed response to the treatment (69). Since the doubling time was not significant, whereas the 5-folding time showed a significant difference, it seems that the antitumor effects of ultrasound in the presence of GNPs provide a delayed response. The best response to the treatment, longer survival time, and the largest lost tissue volume for IPL and ultrasound in the presence of GNPs group were other findings (69). This result shows that the tumor response to treatment appears 24 hr after the initiation of the treatment (69). 

## Conclusion

 Acoustic cavitation in the presence of GNPs and IPL has been introduced as a new method to enhance the therapeutic effects on tumor cells. Due to the low penetration depth of light, this method can be utilized for shallow treatment sites. 

This investigation confirms cavitation in the presence of GNPs and IPL in cancer treatment. This method can be employed to increase the penetration depth by using NPs with an intensive absorption peak in the near infrared region. Such results were also confirmed in other similar investigations on phantoms. One restriction in utilizing the synergy between laser and SDT-mediated GNPs is related to the low penetration depth of photons with 532 nm wavelength. This limitation may be removed by using NIR absorber of NPs along with NIR laser radiation. Future investigations are highly recommended in order to present a more conclusive answer to questions.
